# The Impact of Cefuroxime Susceptibility on *Aeromonas* Necrotizing Fasciitis Outcomes

**DOI:** 10.3390/microorganisms11112776

**Published:** 2023-11-15

**Authors:** Tsung-Yu Huang, Shu-Fang Kuo, Yao-Hung Tsai, Jiun-Liang Chen, Kuo-Ti Peng, Yao-Kuang Huang, Chien-Hui Hung, Yen-Yao Li, Hsing-Jung Li, Cheng-Ting Hsiao, Wei-Hsiu Hsu

**Affiliations:** 1 Division of Infectious Diseases, Department of Internal Medicine, Chiayi Chang Gung Memorial Hospital, Chiayi City 61363, Taiwan; r12045@cgmh.org.tw (T.-Y.H.); hungc01@mail.cgu.edu.tw (C.-H.H.); 2 College of Medicine, Chang Gung University, Taoyuan 33303, Taiwan; orma2244@cgmh.org.tw (Y.-H.T.); yq0139@cgmh.org.tw (J.-L.C.); mr3497@cgmh.org.tw (K.-T.P.); huang137@icloud.com (Y.-K.H.); orthoyao@cgmh.org.tw (Y.-Y.L.); 3 Microbiology Treatment and Research Center, Chiayi Chang Gung Memorial Hospital, Chiayi City 61363, Taiwan; ivykuo@cgmh.org.tw; 4 Departments of Laboratory Medicine, Chiayi Chang Gung Memorial Hospital, Chiayi City 61363, Taiwan; 5 Department of Medical Biotechnology and Laboratory Sciences, College of Medicine, Chang Gung University, Taoyuan 33303, Taiwan; 6 Department of Orthopedic Surgery, Chiayi Chang Gung Memorial Hospital, Chiayi City 61363, Taiwan; 7 Division of Thoracic and Cardiovascular Surgery, Chiayi Chang Gung Memorial Hospital, Chiayi City 61363, Taiwan; 8 Graduate Institute of Clinical Medical Sciences, College of Medicine, Chang-Gung University, Taoyuan 33302, Taiwan; 9 Department of Pediatrics, St. Martin De Porres Hospital, Chiayi City 60069, Taiwan; dongdingkimo@hotmail.com; 10 Department of Emergency Medicine, Chiayi Chang Gung Memorial Hospital, Chiayi City 61363, Taiwan

**Keywords:** necrotizing fasciitis, *Aeromonas*, antimicrobial susceptibility, and minimum inhibitory concentrations

## Abstract

Despite aggressive antibiotic therapy and surgical debridement, *Aeromonas* necrotizing fasciitis (NF) can lead to high amputation and mortality rates. Our study compares the different antibiotic minimum inhibitory concentrations (MICs) via Epsilometer tests (E-tests) between non-survivors and survivors of *Aeromonas* NF of limbs. A prospective review of 16 patients with *Aeromonas* NF was conducted for 3.5 years in a tertiary coastal hospital. E-tests were conducted for 15 antimicrobial agents to determine the MIC value for *Aeromonas* species. These patients were divided into non-survival and survival groups. The clinical outcomes, demographics, comorbidities, presenting signs and symptoms, laboratory findings, and microbiological results between the two periods were compared. A total of four patients died, whereas 12 survived, resulting in a 25% mortality rate. A higher proportion of bloodstream infections (100% vs. 41.7%; *p* = 0.042), monomicrobial infections (100% vs. 33.3%; *p* = 0.021), shock (100% vs. 33.3%; *p* = 0.021), serous bullae (50% vs. 0%; *p* = 0.009), liver cirrhosis (100% vs. 25%; *p* = 0.009), chronic kidney disease (100% vs. 33.3%; *p* = 0.021), lower susceptibility to cefuroxime (25% vs. 83.3%; *p* = 0.028), and ineffective antibiotic prescriptions (75% vs. 16.7%; *p* = 0.029) was observed in non-survivors. *Aeromonas* NF is an extremely rare skin and soft-tissue infection that is associated with high mortality, bacteremia, antibiotic resistance, and polymicrobial infection. Therefore, antibiotic regimen selection is rendered very challenging. To improve clinical outcomes and irrational antimicrobial usage, experienced microbiologists can help physicians identify specific pathogens and test MIC.

## 1. Introduction

Currently, antibiotic resistance remains one of the greatest threats, and *Aeromonas* species are no exception [1,2]. The genus *Aeromonas* is a Gram-negative rod found in freshwater, estuaries, and marine environments [1,2]. Necrotizing fasciitis (NF) caused by *Aeromonas* species is a rare but potentially life-threatening necrotizing skin and soft-tissue infection (NSSTI) characterized by rapidly spreading necrosis, particularly in the fascia [3,4,5]. *Aeromonas* NF has a high mortality (26.4–100%) [3,4,5,6,7] and amputation rate (26.4–50%) despite aggressive surgical and antibiotic treatment [3,4,5,6].

Chia-Yi Chang Gung Memorial Hospital (CGMH) is a tertiary coastal hospital located in West Taiwan. Often, residents of this region encounter raw seafood, seawater, brackish water, and soil. Thus, *Aeromonas* species and *Vibrio* species infections are relatively common [3,4,5,6,7,8,9,10]. Therefore, we set up the *Vibrio* NSSTI Treatment and Research (VTR) Group with professional staff from specialties such as emergency medicine, intensive care unit (ICU), orthopedic surgery, infectious diseases (ID), and plastic surgery, with a hyperbaric oxygen treatment center [5,6,11,12]. A protocol was developed to diagnose and treat NF with broad-spectrum antibiotics with a third-generation cephalosporin plus glycopeptide, and a hospital-wide computerized antimicrobial approval system (HCAAS) was implemented to ascertain appropriate antibiotic use [6].

Microbiologists can perform prompt identification of organisms and pathogen susceptibility patterns. Despite the existence of the VTR Group for many years, microbiologists with extensive experience are still lacking. In clinical microbiology laboratories, a disk-diffusion method for routine antimicrobial susceptibility testing (AST) measures drug efficacy against bacteria. The Epsilometer Test (E-test) is a quantitative method for determining the antimicrobial susceptibility and minimum inhibitory concentrations (MICs) of Gram-negative and Gram-positive aerobic bacteria. In this test device, antibiotic concentrations are measured along an exponential gradient and immobilized on a rectangular plastic strip. Additionally, we collected *Aeromonas* isolates and tested MICs using an E-test during this study period. This study aims to determine whether non-survivors of *Aeromonas* NF have different antibiotic susceptibility results than survivors.

## 2. Materials and Methods

### 2.1. Study Design and Setting

**Figure 1:**
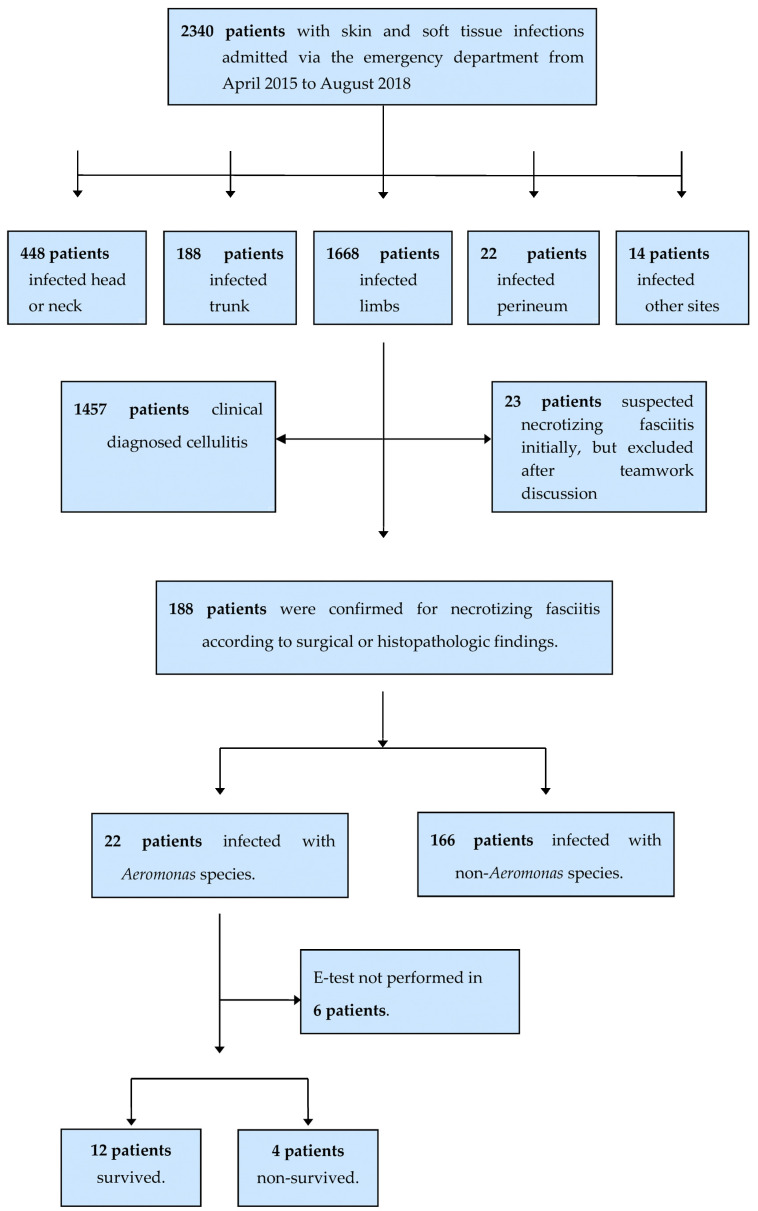
A flowchart showing the clinical isolates of *Aeromonas* strains collected from 16 patients with necrotizing fasciitis.

This is a prospective study of 25 *Aeromonas* species isolated in 16 patients with NF by the VTR Group from April 2015 to August 2018 at our institution. Patients admitted to the emergency department with skin and soft-tissue infections were enrolled. NF of the limbs was established through surgical or histopathologic findings, and *Aeromonas* species were identified using conventional biochemical tests, API-20E Systems, or MALDI-TOF MS. Only patients with *Aeromonas* species infection whose isolates can be re-tested for MIC by an E-test can join this study (Figure 1). Patients were categorized into survival and non-survival groups. The demographics, comorbidities, antimicrobial susceptibility, clinical outcomes, presenting signs and symptoms, and laboratory findings were compared between the two groups.

### 2.2. Definitions

A diagnosis of *Aeromonas* NF is defined as follows: (i) histological or surgical examination findings, such as skin necrosis, subcutaneous fat, superficial fascia, or underlying muscles; (ii) *Aeromonas* spp. was isolated from soft-tissue lesions or blood during an emergency room (ER) visit or surgery [5,11,13]. Empirical antimicrobial regimens were defined to cover all infectious pathogens according to their antimicrobial susceptibilities [9,14]. MIC50 is the MIC that inhibited 50% of bacterial growth, and MIC90 is the MIC that inhibited 90% of bacterial growth.

### 2.3. Laboratory Procedures for Microbiology

The *Aeromonas* species consist of oxidase-positive, polar flagellated, glucose-fermenting, facultatively anaerobic, motile bacteria that do not grow in Gram-negative rods containing 6.5% NaCl. Conventional methods and API-20E Systems (bioMérieux Inc., Hazelwood, MO, USA) were used to identify all strains. For further verification, MALDI-TOF MS (Bruker Daltonik, Bremen, Germany) was used.

### 2.4. Antimicrobial Susceptibility Testing

The disk-diffusion method was used to routinely evaluate all strains for resistance against 10 antimicrobial agents, including cefuroxime, ceftriaxone, ceftazidime, gentamicin, amikacin, ertapenem, ciprofloxacin, levofloxacin, tetracycline, and sulfamethoxazole-trimethoprim. A MIC value for *Aeromonas* species was determined using the E-test method, performed for 15 antimicrobial agents, including ampicillin, ampicillin-sulbactam, cefuroxime, ceftriaxone, ceftazidime, cefepime, gentamicin, amikacin, ertapenem, imipenem, meropenem, ciprofloxacin, levofloxacin, tetracycline, and tigecycline purchased from AB BIODISK (Solna, Sweden) according to the CLSI M45 second edition [15].

### 2.5. Disk-Diffusion Method

Several *Aeromonas* colonies are selected with a sterile inoculating loop. The organism is suspended in 2 mL of Tryptic Soy Broth (TSB). A smooth suspension is created by vortexing the TSB tube. To match a 0.5 McFarland standard, turbidity is adjusted visually using TSB. Within 15 min of preparation, this suspension is used. A sterile cotton swab is placed in the inoculum tube. The dried surface of an MH agar plate is inoculated by streaking the swab three times across the entire agar surface. Disks are applied to the agar surface with a dispenser and then incubated for 16–18 h at 35℃ in an ambient air incubator. The inhibition zone diameters are then measured to interpret the susceptibility test results according to the CLSI M45 second edition.

### 2.6. E-Test

Several *Aeromonas* colonies are selected with a sterile inoculating loop. The organism is suspended in 2 mL TSB. A smooth suspension is created by vortexing the TSB tube. To match a 0.5 McFarland standard, turbidity is adjusted visually using TSB. Within 15 min of preparation, this suspension is used. A sterile cotton swab is placed in the inoculum tube. The dried surface of an MH agar plate is inoculated by streaking the swab three times across the entire agar surface. The strip is then placed with the “E end” at the edge of the plate and with the scale visible. Plates are then incubated at 35 °C for 16–20 h. The susceptibility test results are interpreted according to the CLSI M45 2nd edition.

### 2.7. Statistical Analysis

To determine predictors of non-survivors, a logistic regression model analysis was conducted. Chi-square tests were used to test categorical variables, and Student’s *t*-tests and ANOVA were used to test continuous variables. Statistical significance was determined by *p*-values < 0.05 with two-tailed tests. Statistical analysis was conducted using SPSS version 25.0 for Windows (Chicago, IL, USA).

## 3. Results

### 3.1. Patient Selection and Clinical Isolates

From April 2015 to August 2018, 22 patients admitted via the ER were diagnosed with *Aeromonas* NF of the limbs (Figure 1). Furthermore, 25 *Aeromonas* isolates were collected from 16 patients. We obtained 10 isolates from wounds, nine from blood, four from tissue, and two from pus. The MICs of *Aeromonas* strains isolated from 16 patients with NF of the limbs are shown in Table 1. Table 2 shows susceptibility, intermediate status, and resistance for 16 *Aeromonas* NF patients as determined by the disk-diffusion and E-test methods, as well as the MIC50, MIC90, and MIC range using the E-test method. Among the *Aeromonas* species, ampicillin resistance is the highest (50%), followed by cefuroxime (25%), using the E-test method. However, in the disk-diffusion method, only 12.5% showed cefuroxime resistance.

### 3.2. Microbiological Analysis and Empiric Antibiotics

*Aeromonas hydrophila* accounted for 14 (87.5%) of the 16 *Aeromonas* NF patients, followed by one *Aeromonas caviae* (6.25%) and one *Aeromonas veronii* biovar *sobria* (6.25%). The data for 16 cases of *Aeromonas* necrotizing fasciitis are summarized in Table 3.

The non-survival and survival group included 4 and 12 patients, respectively, resulting in a 25% mortality rate. Additionally, we found a higher proportion of bloodstream (100% vs. 41.7%; *p* = 0.042) and monomicrobial infections (100% vs. 33.3%; *p* = 0.021) in non-survivors (Table 4). In the *Aeromonas* susceptibility to cefuroxime, the non-survival group had a statistically significant lower susceptibility (25% vs. 83.3%; *p* = 0.028). In the ER, the non-survival group had a higher rate of ineffective antibiotic prescriptions (75% vs. 16.7%; *p* = 0.029) (Table 4). Three non-survivals were prescribed ceftriaxone at the ER, two were changed to ciprofloxacin because of their resistance, and one was re-adjusted to levofloxacin because of the co-infection of pneumonia and respiratory failure. Based on antibiotic susceptibility, two survivors were prescribed cefazolin at the ER and escalated to cefuroxime.

### 3.3. Data on Demographics, Characteristics, and Clinical Outcomes

Patients in the non-survival group required more ICU care (100% vs. 41.7%; *p* = 0.042) and postoperative intubation (100% vs. 25%; *p* = 0.009) and had higher rates of alcoholism (100% vs. 41.7%; *p* = 0.042), liver cirrhosis (100% vs. 25%; *p* = 0.009), and chronic kidney disease (100% vs. 33.3%; *p* = 0.021) than those in the survival group. Non-survival patients had a statistically longer ICU stay (days) (18.3 ± 15.0 vs. 1.1 ± 1.8; *p* = 0.001) (Table 5).

### 3.4. Surgical Treatment

As for the first surgery, 14 (87.5%) and two (12.5%) patients received fasciotomy and debridement, respectively (Table 5). Between the two groups, no difference was found in the first surgical method and the number of surgical operations or amputations.

### 3.5. Clinical Presentations

No significant differences between the two groups were found in the symptom and sign duration, presence of fever (body temperature > 38 °C), tachycardia (heart rate > 100/min), or tachypnea (respiratory rate > 20/min). Also, no difference was found between the two groups in the proportion of patients with erythematous, swollen, painful lesions, hemorrhagic bullae, and skin necrosis. The proportion of patients presenting with shock (systolic blood pressure < 90 mmHg, 100% vs. 33.3%; *p* = 0.021) and serous bullae (50% vs. 0%; *p* = 0.009) was higher in the non-survival groups (Table 6).

### 3.6. Laboratory Findings

Hemoglobin < 10 g/dL was detected more frequently in the non-survival group (Table 7). Also, the non-survival group had a longer prothrombin time (*p* < 0.001), activated partial thromboplastin time (*p* = 0.002), and higher serum total bilirubin (*p* = 0.005).

## 4. Discussion

In the family *Aeromonadaceae* of Gram-negative bacteria, aeromonads are rod-shaped, facultatively anaerobic, nonsporulating bacteria adapted to aquatic environments [1], including freshwater, seawater, sewage, and freshwater junctions. Currently, three major species are recognized as human pathogens: *Aeromonas hydrophila*, *Aeromonas caviae*, and *Aeromonas veronii* biovar *sobria* [1,16]. Conventional biochemical fermentation tests can only identify specific types of bacteria based on their metabolism, and they may not be suitable for identifying all bacterial strains. Ambiguous results can arise when different genera yield the same outcome. A total of five (31.3%) isolates from 16 patients were not able to be identified to species using conventional methods and API-20E systems. MALDI-TOF MS was used for further verification, and all isolates were successfully identified. In healthy participants, these bacteria can cause diarrhea, biliary tract infections, bloodstream infections, and skin and soft-tissue infections [5,6,17,18,19,20,21,22]. Immunocompromised patients, such as those with chronic liver disease, chronic kidney disease, diabetes mellitus, or malignant disease, are more susceptible to NF [4,5,13,22].

NF is a rare and life-threatening NSSTI [23]. Among the monomicrobial Gram-negative NF, *Vibrio* species and *Aeromonas* species are the most prevalent and fulminant pathogens [5,6,7,8,9,11,12,24]. *Aeromonas* bacteremia has a fatality rate of 30–70% [17,18,22]. *Aeromonas* NF can cause limb loss or even death if not diagnosed early and treated appropriately [3,4,5,6,7]. *Aeromonas* SSTIs are often polymicrobial [5,6,19] and exhibit high drug resistance [5,6], rendering it difficult to select an appropriate antimicrobial regimen. Despite aggressive surgical debridement and antimicrobial usage, initial ineffective antimicrobial treatments for *Aeromonas* NF still lead to high mortality [5,6]. NF diagnosis and treatment protocols were established by our VTR Group [6]. In Taiwan, HCAAS was implemented by the CGMH, which is an online antimicrobial control system [6,25,26]. HCAAS has been used by an experienced ID physician in CGMH-Chiayi to improve rational antibiotic use and clinical outcomes in ICU trauma and *Aeromonas* NF patients [6,26].

Before prescribing antibiotics, physicians should conduct infectious pathogen identification and AST. In vitro, ASTs can be performed accurately, reproducibly, and timely in clinical laboratories to ascertain clinical relevance. Qualitative disk diffusion, which is a traditional classification method [27], measures the inhibition zone diameter and quantitative dilution methods (broth or agar dilutions) [28], including the performance of the E-test [29,30], which determines the MIC of antibiotics. In microbiology laboratories, MICs are used to confirm microbe resistance [31,32]. Most clinical microbiology laboratories routinely test antimicrobial susceptibility using agar disk-diffusion testing, which was developed in 1940 [27]. Zones of inhibition are measured around paper disks containing antibiotics on agar culture dishes that have been evenly inoculated with bacteria. Antibiograms categorize bacteria as susceptible, intermediate, or resistant [33]. The advantages of disk-diffusion assays include simplicity, cost-efficiency, and the ability to test large numbers of microbes and antimicrobial agents. However, the agar disk-diffusion method is unsuitable for determining the MIC since it is impossible to quantify the amount of antimicrobial agent diffused into the medium.

In the past, reports have elucidated that antimicrobial resistance may develop during *Aeromonas* treatment [6,18]. As a second method to test drug susceptibility for the first isolated *Aeromonas* species, an E-test was used to re-evaluate the AST results. By placing a strip impregnated with antimicrobials on an agar plate, an E-test, an antimicrobial gradient method, can determine the MIC value. The procedure involves depositing an increasing concentration gradient of the antimicrobial agent on an agar surface inoculated with the microorganism being tested. A MIC value is determined when the strip intersects with the growth inhibition ellipse. However, E-test strips cost at least $2–3 (US dollars) each; therefore, this approach can be costly if numerous antibiotics are tested [33].

Resistance to penicillin and ampicillin is demonstrated by a majority of *Aeromonas* strains, while most are invariably susceptible to sulfa drugs, second- to fourth-generation cephalosporins, and carbapenems [34,35]. Aminoglycosides, fluoroquinolones, tetracyclines, and tigecycline are usually effective against the *Aeromonas* species [34,35,36]. Our study results are consistent with previous studies. The initial ineffective empirical antimicrobial usage is related to poor outcomes in patients with *Aeromonas* NF [5,6]. *Aeromonas* NF, which is cephalosporin-resistant, can have a mortality rate of 40% [5]. Culture-directed antimicrobial therapy should be aggressively administered to prevent antibiotic use delay in critically ill patients [7,26]. Ordering antibiotics with culture and susceptibility results is more appropriate than requesting them empirically [26]. In ER, oxacillin and gentamicin were prescribed empirically for NF infections before 2006, and third-generation cephalosporins were prescribed empirically for *Vibrio* and *Aeromonas* infections after 2006 [6]. Through HCAAS, an experienced ID physician can confirm an effective antimicrobial regimen against *Aeromonas* species [6]. Overall, 68.75% of *Aeromonas* species were susceptible to the disk-diffusion method, and in this study, cefuroxime susceptibility (25%) was significantly lower in the non-survival group. In this study, we found a two-fold cefuroxime resistance between the E-test and disk-diffusion methods. Therefore, after fulminant *Aeromonas* NF is established, re-testing an E-test for cefuroxime, ceftriaxone, and ertapenem should be conducted. Usually, these antibiotics have higher resistance rates and are used empirically; however, they are easily underestimated through disk diffusion.

According to a 30-year study, patients with bacteremia, septic shock, polymicrobial bacteremia, and inappropriate empirical antimicrobial treatment have a high risk of mortality [37]. *Aeromonas* NF combined with bloodstream infection significantly increased mortality [5,38]. It is estimated that 36.1–50% of deaths are caused by *Aeromonas* SSTI combined with bloodstream infection [5,6,21]. Conversely, monomicrobial *Aeromonas* NF infections are significantly more likely to result in death than polymicrobial infections [5]. In our study, all nonsurvivors had monomicrobial infections with *Aeromonas* and had bloodstream infections in addition to their NF.

Approximately 36–100% of patients with NF require ICU care [5,6,12,39] and 17–61% require postoperative intubation [6,12,39]. All non-survival patients in this study require ICU care and postoperative intubation. Patients with NF who also had chronic kidney disease, chronic liver dysfunction, diabetes mellitus, hematological diseases, or malignancy had a significantly higher mortality rate [4,5,13,21,38]. Approximately 27–33% of patients with *Aeromonas* NF also exhibited hepatic cirrhosis [4,5,6]. Compared with survivors, non-survivors had a higher rate of alcoholism (100%), liver cirrhosis (100%), chronic kidney disease (100%), and prolonged ICU stays.

The non-survival group exhibited a statistical tendency to manifest septic shock, anemia, and hyperbilirubinemia more than the survival group [5,38]. Some of the literature has reported that initial presentations of tachycardia and tachypnea were also predictors of poor outcomes in patients with NF [5,38]. In this study, neither tachycardia nor tachypnea were observed in the non-survival group, which may be attributed to the small sample size. *Aeromonas* NF patients frequently presented with hemorrhagic bullae and skin necrosis [3,4,5,7,12]. The mortality rate of patients with NF presenting with hemorrhagic bullae is higher than that of patients with serous-filled bullae or without bullae [12]. Skin necrosis was present in 28% of patients with *Aeromonas* NF, which was also a poor predictor of mortality [5,6]. Serous bullae were found in 12.5% of the patients. More patients in the non-survival group had more serous bullae than the survivor group. This is the first time *Aeromonas* NF has been reported with such an early clinical finding. In critically ill patients with fulminant NF, prompt fasciotomy combined with appropriate empiric antimicrobial therapy and the use of HCAAS by an experienced ID physician has been proven to save lives and limbs [6,7,13,26].

It has been reported that plasmid-encoded AmpC β-lactamases frequently cause β-lactam resistance in clinical strains. There are AmpC enzymes in the chromosomes of several bacterial genera, including *Aeromonas*. The ampC gene of *Aeromonas* may increase its responsiveness to antibiotic selection pressure [40]. AQU-1 and blaMOX, which are both Amp-C antibiotics, were detected in all *Aeromonas caviae* isolates and in 91.9% of *Aeromonas dhakensis* isolates, according to previous reports. Tigecycline, fluoroquinolones, and cefepime showed good anti-aeromonad activity in vitro [41]. Hospital-associated infections caused by carbapenemase-producing *Aeromonas* can be fatal. Despite being treated with multiple broad-spectrum antibiotic regimens to overcome various carbapenemase classes, these patients died from underlying comorbidities [42]. Several resistance determinants were found, including co-occurring KPC-3 and VIM-2, OXA-232, and chromosomal CphA-like carbapenemase genes, resulting in major treatment challenges [42].

This study has several limitations. The study was limited to only 16 patients, and 15 antimicrobial agents were tested using an E-test for MIC. Second, this study period was performed for only 3.5 years. Third, no AST test for *Aeromonas* species during treatment was performed during follow-up.

## 5. Conclusions

*Aeromonas* NF is a very rare NSSTI with high mortality, antibiotic resistance, and polymicrobial composition, rendering antibiotic treatment very challenging. AST performed by experienced microbiologists can identify the true pathogen causing NF and determine the appropriate antibiotic to be used.

## Figures and Tables

**Table 1:** MIC ^a^ of *Aeromonas* strains isolated from 16 patients with necrotizing fasciitis of limbs to different antimicrobial agents.

Case No.	AMP	SAM	CXM	CRO	CAZ	FEP	GM	AN	ETP	IMP	MEM	CIP	LVX	TC	TGC
**1**	128	16	1	1	0.064	0.032	0.19	0.75	4	4	0.032	0.004	0.012	0.75	0.032
**2**	256	16	0.75	0.5	0.096	0.047	0.25	2	4	4	0.032	0.008	0.016	1.5	1
**3**	2	2	3	0.023	0.19	1	0.125	1.5	0.008	0.5	0.047	0.094	0.25	0.5	0.064
**4**	256	32	1	0.5	0.064	0.047	0.19	2	0.125	0.38	0.094	4	0.38	24	0.38
**5**	256	64	1	1	0.125	0.094	0.19	1.5	8	0.38	0.032	0.006	0.016	1	0.5
**6**	256	24	1.5	1	0.19	0.064	0.25	1.5	0.38	0.38	0.094	0.006	0.016	1	0.38
**7**	1	1	2	0.016	0.19	0.5	1	4	0.002	0.5	0.012	0.016	0.032	1	0.032
**8**	1.5	2	2	0.023	0.19	1	0.25	1.5	4	0.5	0.047	0.125	0.19	0.5	0.064
**9**	2	1.5	32	4	0.125	1	0.125	1.5	0.008	0.75	0.032	0.125	0.25	0.5	0.047
**10**	256	24	1.5	0.023	0.19	0.094	0.25	1.5	0.008	0.5	0.047	0.125	0.032	0.5	0.047
**11**	256	2	32	4	16	0.5	0.38	1.5	0.38	0.5	0.047	0.006	0.19	0.5	0.38
**12**	2	2	1	1	0.125	0.5	0.125	1.5	0.008	0.75	0.032	0.125	0.25	0.5	0.047
**13**	2	2	3	0.023	0.19	1	0.125	1.5	0.38	0.75	0.047	0.006	0.25	0.5	0.38
**14**	2	1.5	16	0.032	0.125	1	0.125	1.5	0.008	0.5	0.032	0.125	0.25	0.5	0.047
**15**	2	2	3	0.023	0.19	1	0.38	1.5	0.008	0.75	0.047	0.125	0.25	0.5	0.047
**16**	256	64	32	4	16	1	8	1.5	0.008	0.75	0.047	0.125	0.25	16	0.047

Abbreviations: ^a^ MIC: minimum inhibitory concentration; AMP: ampicillin; SAM: ampicillin-sulbactam; CXM: cefuroxime; CRO: ceftriaxone; CAZ: ceftazidime; FEP: cefepime; GM: gentamicin; AN: amikacin; ETP: ertapenem; IMP: imipenem; MEM: meropenem; CIP: ciprofloxacin; LVX: levofloxacin; TC: tetracycline; TGC: tigecycline.

**Table 2:** Susceptibilities of *Aeromonas* strains isolated from 16 patients by CLSI ^a^ breakpoint interpretation.

Antimicrobial Agents	MIC Breakpoint (μg/mL)			MIC (ug/mL)		E-Test (%)			Zone Diameter Breakpoint (mm)			Disk Diffusion (%)		
	S	I	R	MIC50 ^b^	MIC90 ^c^	S	I	R	S	I	R	S	I	R
Ampicillin	≤8	16	≥32	128	256	50		50	≥17	14–16	≤13			
Unasyn ^d^	≤8/4	2	≥32/16	2	32	56.3	25	18.8	≥15	12–14	≤11			
Cefuroxime	≤1	2	≥4	2	32	68.8	6.3	25	≥26	23–25	≤22	75	12.5	12.5
Ceftriaxone	≤1	2	≥4	0.032	4	81.3		18.8	≥23	20–22	≤19	81.3		18.8
Ceftazidime	≤4	8	≥16	0.125	0.19	87.5		12.5	≥21	18–20	≤17	87.5		12.5
Cefepime	≤8	16	≥32	0.5	1	100			≥18	15–17	≤14			
Gentamicin	≤4	8	≥16	0.19	1	93.8	6.3		≥15	13–14	≤12	93.8		6.3
Amikacin	≤16	32	≥64	0.75	2	100			≥17	15–16	≤14	100		
Ertapenem	≤2	4	≥8	0.125	4	75	12.5	12.5	≥19	16–18	≤15	68.8	18.8	12.5
Imipenem	≤4	8	≥16	0.5	0.75	87.5		12.5	≥16	14–15	≤13			
Meropenem	≤4	8	≥16	0.032	0.047	100			≥16	14–15	≤13			
Ciprofloxacin	≤1	2	≥4	0.016	0.125	93.8		6.3	≥21	16–20	≤15	93.8		6.3
Levofloxacin	≤2	4	≥8	0.19	0.25	100			≥17	14–16	≤13	100		
Tetracycline	≤4	8	≥16	0.5	16	87.5		12.5	≥15	12–14	≤11	87.5		12.5
Tigecycline	≤2	4	≥8	0.047	0.5	100								
TMP-SMX ^e^	≤2/38	-	≥4/76						≥16	11–15	≤10	81.3		18.8

Abbreviations: ^a^ CLSI: Clinical and Laboratory Standards Institute; ^b^ MIC50: minimum inhibitory concentrations that inhibited 50% of the isolates; ^c^ MIC90: minimum inhibitory concentrations that inhibited 90% of the isolates; ^d^ Unasyn: Ampicillin-sulbactam; ^e^ TMP-SMX: Trimethoprim-Sulfamethoxazole. S, I, and R stand for susceptible, intermediate, and resistant, respectively.

**Table 3:** Summary of data for 16 cases of *Aeromonas* necrotizing fasciitis.

Case No.	Age/ Gender	Underlying Disease	Antibiotic Regimen at ER ^a^	First Types of Surgery	Outcome
1	63/M	Hepatitis B	Ceftriaxone, vancomycin	Fasciectomy	Recovered
2	64/M	CKD ^b^ under H/D ^c^, DM ^d^, hepatitis C	Ceftriaxone	Debridement	Recovered
3	57/M	Alcoholism, DM, hepatitis C	Ceftriaxone, doxycycline	Fasciectomy	Recovered
4	76/M	DM, lymphoma, prostate cancer	Ceftriaxone, vancomycin	Fasciectomy	Recovered
5	46/M	Alcoholism, DM, liver cirrhosis	Ceftriaxone, vancomycin, metronidazole	Fasciectomy	Recovered
6	77/M	Not evident	Ceftriaxone, teicoplanin, metronidazole	Fasciectomy	Recovered
7	49/M	Alcoholism, CKD	Ceftriaxone, teicoplanin, doxycycline	Fasciectomy	Recovered
8	63/M	Alcoholism, DM	Cefazolin, doxycycline	Fasciectomy	Recovered
9	52/M	Alcoholism, CKD, DM, hepatitis B, liver cirrhosis	Ceftriaxone, doxycycline	Fasciectomy	Died
10	58/M	Not evident	Ceftriaxone, doxycycline	Fasciectomy	Recovered
11	65/F	DM, hepatitis C	Ceftriaxone, metronidazole, doxycycline	Fasciectomy	Recovered
12	54/M	Alcoholism, CKD under H/D, DM, hepatoma, liver cirrhosis	Ceftriaxone, metronidazole, doxycycline	Fasciectomy	Died, amputation
13	47/M	Alcoholism, CKD under H/D, DM, hepatitis B, hepatoma, liver cirrhosis	Ceftriaxone, teicoplanin, doxycycline	Fasciectomy	Died
14	79/M	Colon cancer, CKD, DM, hepatitis B & C, liver cirrhosis	Ceftriaxone, doxycycline	Fasciectomy	Recovered
15	79/M	Alcoholism, CKD, hepatitis C, liver cirrhosis	Ceftriaxone, amikacin, doxycycline	Fasciectomy	Recovered
16	59/M	Alcoholism, CKD under H/D, liver cirrhosis	Ceftriaxone	Debridement	Died

Abbreviations: ^a^ ER: emergency room; ^b^ CKD: chronic kidney disease; ^c^ H/D: hemodialysis; ^d^ DM: Diabetes mellitus.

**Table 4:** Microbiological results, antimicrobial susceptibilities, and antimicrobial usage for 16 patients between non-survival and survival groups.

Variable	Non-Survival (*n* = 4)	Survival (*n* = 12)	*p*-Value
Bloodstream infection	4 (100)	5 (41.7)	0.042 *
Monomicrobial infection	4 (100)	4 (33.3)	0.021 *
Susceptibility (MIC interpretation)			
Ampicillin	3 (75)	5 (41.7)	0.248
Ampicillin-sulbactam	3 (75)	6 (50)	0.411
Cefuroxime	1 (25)	10 (83.3)	0.028 *
Ceftriaxone	2 (50)	11 (91.6)	0.064
Ceftazidime	3 (75)	11 (91.6)	0.383
Cefepime	4 (100)	12 (100)	-
Gentamicin	3 (75)	12 (100)	0.074
Amikacin	4 (100)	12 (100)	-
Ertapenem	4 (100)	8 (66.7)	0.411
Imipenem	4 (100)	10 (83.3)	0.383
Meropenem	4 (100)	12 (100)	-
Ciprofloxacin	4 (100)	11 (91.6)	0.551
Levofloxacin	4 (100)	12 (100)	-
Tetracycline	3 (75)	11 (91.6)	0.383
Tigecycline	4 (100)	12 (100)	-
Colistin	0 (0)	5 (41.7)	0.126
Ineffective empirical antimicrobial usage at ER	3 (75)	2 (16.7)	0.029 *

Data were presented as frequency (%). *: *p*-value < 0.05.

**Table 5:** Demographic data, comorbidities, and clinical outcomes of 16 patients between non-survival and survival groups.

Variable	Non-Survival (*n* = 4)	Survival (*n* = 12)	*p*-Value
Age (years)	53.0 ± 5.0	64.7 ± 11.3	0.069
Sex, male	4 (100)	11 (91.6)	0.551
Involved region			0.182
Lower extremities	4 (100)	8 (66.7)	
Disease severity			
APACHE ^a^ II score	20.0 ± 5.4	14.3 ± 5.9	0.109
ICU ^b^ stay	4 (100)	5 (41.7)	0.042 *
Postoperative intubation	4 (100)	3 (25)	0.009 *
Underlying chronic diseases			
Chronic liver dysfunction	4 (100)	9 (75)	0.267
Alcoholism	4 (100)	5 (41.7)	0.042 *
Viral hepatitis	2 (50)	6 (50)	1.000
Liver cirrhosis	4 (100)	3 (25)	0.009 *
Chronic kidney disease	4 (100)	4 (33.3)	0.021 *
Cancer	2 (50)	2 (16.7)	0.182
Diabetes mellitus	3 (75)	7 (58.3)	0.551
Misdiagnosis at ER	1 (25)	2 (16.7)	0.712
Methods of the first operation			0.383
Fasciectomy	3 (75)	11 (91.6)	
Debridement	1 (25)	1 (8.3)	
Number of surgical operations	2.5 ± 1.7	2.8 ± 1.4	0.769
Clinical outcomes			
Number of amputations	1 (25)	0 (0)	0.074
ICU stay (days)	18.3 ± 15.0	1.1 ± 1.8	0.001 *
Hospital stays (days)	19.0 ± 17.3	35.9 ± 15.4	0.086

Data were presented as mean (standard deviation) or frequency (%). *: *p*-value < 0.05. ^a^ APACHE: Acute Physiology and Chronic Health Evaluation, ^b^ ICU: intensive care unit.

**Table 6:** Clinical presentations of 16 patients with *Aeromonas* necrotizing fasciitis between non-survival and survival groups.

Variable	Non-Survival (*n* = 4)	Survival (*n* = 12)	*p*-Value
Duration of symptoms/signs (days)	1.5 ± 1.0	1.5 ± 0.8	1.000
Symptoms/signs of systemic disease			
Shock ^a^	4 (100)	4 (33.3)	0.021 *
Tachycardia ^b^	4 (100)	9 (75.0)	0.276
Tachypnea ^c^	2 (50)	5 (41.7)	0.771
Fever ^d^	1 (25)	3 (25)	1.000
Symptoms/signs of the limbs			
Pain and tenderness	4 (100)	11 (91.6)	0.551
Swelling and erythema	4 (100)	9 (75.0)	0.267
Hemorrhagic bullae	2 (50)	4 (33.3)	0.551
Serous bullae	2 (50)	0 (0)	0.009 *
Skin necrosis	2 (50)	1 (8.3)	0.064

Data were presented as mean (standard deviation) or frequency (%).*: *p*-value < 0.05. ^a^ Shock: systolic blood pressure < 90 mmHg; ^b^ Tachycardia: heartbeat > 100/min; ^c^ Tachypnea: respiratory rate > 20/min; ^d^ Fever: body temperature > 38 °C.

**Table 7:** Laboratory findings of 16 patients with *Aeromonas* necrotizing fasciitis between non-survival and survival groups.

Variable	Non-Survival (*n* = 4)	Survival (*n* = 12)	*p*-Value
Total WBC ^a^ count			
Leukocytosis (≥12,000/μL)	1 (25)	9 (75)	0.074
Leukopenia (≤4000/μL)	1 (25)	1 (8.3)	0.383
Differential count			
Neutrophilia (>7500/μL)	2 (50)	9 (75)	0.350
Band forms (>10%)	2 (50)	4 (33.3)	0.551
Lymphocytopenia (<1000/μL)	4 (100)	7 (58.3)	0.119
Hemoglobin < 10 g/dL	3 (75)	1 (8.3)	0.008 *
Thrombocytopenia (platelet count < 15 × 10^4^/μL)	4 (100)	6 (50)	0.074
eGFR ^b^ < 30 c.c./min	3 (75)	3 (25)	0.074
C-reactive protein (mg/dL)	93.8 ± 69.5	108.2 ± 124.1	0.831
Albumin (mg/dL)	2.2 ± 0.8	3.2 ± 0.9	0.054
PT ^c^ (s)	25.2 ± 9.4	11.9 ± 2.0	<0.001 *
APTT ^d^ (s)	62.7 ± 23.7	32.2 ± 8.2	0.002 *
Total bilirubin (mg/dL)	6.0 ± 2.6	1.8 ± 1.7	0.005 *

Data were presented as mean (standard deviation) or frequency (%).*: *p*-value < 0.05. Abbreviations: ^a^ WBC: white blood cell; ^b^ eGFR: estimated glomerular filtration rate; ^c^ PT: prothrombin time; ^d^ APTT: activated partial thromboplastin time.

## Data Availability

The datasets analyzed during the current study are not publicly available, due to confidentiality reasons, but are available from the corresponding author upon reasonable request.

## Abbreviations

NF, necrotizing fasciitis; E-test, Epsilometer test; MIC, minimum inhibitory concentration; NSSTI, necrotizing skin, and soft-tissue infection.
